# A phase II study to evaluate LY2603618 in combination with gemcitabine in pancreatic cancer patients

**DOI:** 10.1186/s12885-017-3131-x

**Published:** 2017-02-15

**Authors:** Berta Laquente, Jose Lopez-Martin, Donald Richards, Gerald Illerhaus, David Z. Chang, George Kim, Philip Stella, Dirk Richel, Cezary Szcylik, Stefano Cascinu, G. L. Frassineti, Tudor Ciuleanu, Karla Hurt, Scott Hynes, Ji Lin, Aimee Bence Lin, Daniel Von Hoff, Emiliano Calvo

**Affiliations:** 1 Institut Català d’Oncologia-IDIBELL (Institut d’Investigació Biomèdica de Bellvitge), Barcelona, Spain; 2 University Hospital and Research Institute, Madrid, Spain; 3 US Oncology Research, Tyler, USA; 4 Hematology, Onkology, and Palliative Care, Klinikum Stuttgart, Stuttgart, Germany; 5 Virginia Oncology Associates, Eastern Virginia Medical School, US Oncology Research, Hampton, VA USA; 6 21st Century Oncology, University of Florida Health Oncology, Jacksonville, USA; 7 St. Joseph Mercy Hospital, Ypsilanti, MI USA; 8 Academic Medical Center, Amsterdam, Netherlands; 9 Department of Oncology, Military Institute of Medicine, Warsaw, Poland; 10 Department of Oncology and Hematology, Universitá di Modena e Reggio Emilia, Policlinico di Modena, Modena, Italy; 11 Department of Oncology, Istituto Scientifico Romagnolo per lo Studio e la Cura dei Tumori (IRST) IRCCS, Meldola, Italy; 12 Institute of Oncology Ion Chiricuta, University of Medicine and Pharmacy Iuliu Hatieganu, Cluj Napoca, Romania; 13 Eli Lilly and Company, Indianapolis, IN USA; 14 Translational Genomics Research Institute (TGen) and HonorHealth Research Institute, Phoenix, AZ USA; 15 START Madrid-CIOCC, Centro Integral Oncológico Clara Campal, Medical Oncology Division, Hospital Universitario Madrid Norte Sanchinarro, Calle Oña, 10, 28050 Madrid, Spain

**Keywords:** CHK1, cancer, gemcitabine, phase II, LY2603618

## Abstract

**Background:**

The aim of this study was to determine whether checkpoint kinase 1 inihibitor (CHK1), LY2603618, and gemcitabine prolong overall survival (OS) compared to gemcitabine alone in patients with unresectable pancreatic cancer.

**Methods:**

Patients with Stage II-IV locally advanced or metastatic pancreatic cancer were randomized (2:1) to either 230 mg of LY2603618/1000 mg/m^2^ gemcitabine combined or 1000 mg/m^2^ gemcitabine alone. OS was assessed using both a Bayesian augment control model and traditional frequentist analysis for inference. Progression-free survival (PFS), overall response rate (ORR), duration of response, pharmacokinetics (PK), and safety (Common Terminology Criteria for Adverse Events [AEs] v 3.0) were also evaluated.

**Results:**

Ninety-nine patients (*n* = 65, LY2603618/gemcitabine; *n* = 34, gemcitabine) were randomized (intent-to-treat population). The median OS (months) was 7.8 (range, 0.3–18.9) with LY2603618/gemcitabine and 8.3 (range, 0.8-19.1+) with gemcitabine. Similarly, in a Bayesian analysis, the study was not positive since the posterior probability that LY2603618/gemcitabine was superior to gemcitabine in improving OS was 0.3, which did not exceed the prespecified threshold of 0.8. No significant improvements in PFS, ORR, or duration of response were observed. Drug-related treatment-emergent AEs in both arms included nausea, thrombocytopenia, fatigue, and neutropenia. The severity of AEs with LY2603618/gemcitabine was comparable to gemcitabine. The LY2603618 exposure targets (AUC_(0-∞)_ ≥21,000 ng∙hr/mL and C_max_ ≥2000 ng/mL) predicted for maximum pharmacodynamic response were achieved after 230 mg of LY2603618.

**Conclusions:**

LY2603618/gemcitabine was not superior to gemcitabine for the treatment of patients with pancreatic cancer.

**Trial Registration:**

NCT00839332. Clinicaltrials.gov. Date of registration: 6 February 2009

## Background

Pancreatic cancer is the fourth leading cause of cancer-related deaths in the United States [1]. Current therapeutic strategies for pancreatic cancer have a modest impact on disease course and prognosis [2]. The 5-year survial rate remains low (<5%) [3]. Until recently, gemcitabine was the standard of care for patients with advanced/metastatic pancreatic cancer. FOLFIRINOX (oxaliplatin, irinotecan, leucovorin, and 5-FU) and gemcitabine plus *nab*-paclitaxel (Abraxane®) are novel therapeutic regimens demonstrating survival advantages in patients with advanced pancreatic cancer [4–8]. Although these recent advances are promising, there is still a need for novel therapeutic targets to further improve and sustain clinical response in pancreatic cancer patients.

Checkpoint kinase 1 (CHK1) is a protein kinase involved in the DNA damage response. Activation of CHK1 initiates cell cycle arrest allowing for DNA repair and replication. Inhibition of CHK1 allows cells to enter mitosis without DNA repair, eventually leading to apoptosis [9]. Furthermore, inhibition of CHK1 sensitizes tumor cells to DNA-damaging agents making CHK1 a unique target for cancer therapy. Azorsa and colleagues recently identified CHK1 as a therapeutic target for sensitizing pancreatic cancer cells to gemcitabine therapy using a synthetic lethal RNAi screening approach [10].

LY2603618, a selective CHK1 inhibitor, enhances the activity of cytotoxic chemotherapy agents, including gemcitabine, in in vitro and in vivo nonclinical efficacy studies [11, 12]. Phase I of this Phase I/II study determined the recommended Phase II dose to be 230 mg [13]. Phase II, as presented here, determined if the overall survival (OS) in patients with Stage II-IV unresectable pancreatic cancer who were administered LY2603618 and gemcitabine exceeded the OS of patients treated with gemcitabine alone.

## Methods

### Study objectives

The primary objective of this Phase II study was to compare OS with LY2603618/gemcitabine to gemcitabine alone in patients with Stage II-IV unresectable pancreatic cancer. Secondary objectives included characterizing the safety and toxicity profile of LY2603618/gemcitabine and gemcitabine; estimating progression-free survival (PFS), duration of response, and change in tumor size; assessing response rates; evaluating the pharmacokinetics (PK) of LY2603618; investigating biomarker responses; and performing an exploratory assessment of Fridericia’s heart rate-corrected QT interval (QTcF).

### Patients

Adult patients who had given informed consent had adequate hematological, liver, and renal functions; histological or cytological evidence of a diagnosis of Stage II or III adenocarcinoma of the pancreas not amenable to resection with curative intent or Stage IV disease; and an Eastern Cooperative Oncology Group performance status (ECOG PS) 0–2. Patients with previous radical surgery for pancreatic cancer were eligible after progression was documented. Exclusion criteria included known hypersensitivity to gemcitabine; females who were pregnant or lactating; prior radiotherapy involving >25% of marrow-producing area; and treatment with any non-approved drug within 30 days of enrollment. Patients may have received previous adjuvant treatment with gemcitabine.

### Study design and treatment plan

Prior to enrollment, the study protocol, patient informed consent, and any other written study documentation were approved by an ethics committee. This trial was conducted in accordance with the Declaration of Helsinki and the Good Clinical Practice Guidelines of the International Conference on Harmonization. Phase II of this open-label, multicenter, randomized, 2-arm, Phase I/II trial was conducted in patients with locally advanced or metastatic pancreatic cancer. Patients were randomized (2:1) to either LY2603618/gemcitabine or gemcitabine. Gemcitabine (1000 mg/m^2^) was given as a 30-min infusion on days 1, 8, and 15 of a 28-day cycle. LY2603618 (230 mg) was administered as a 1-h infusion ~24 h after administration of gemcitabine. Patients continued on treatment until disease progression, unacceptable toxicity, or patient unwillingness to participate.

### Statistical analysis

The primary objective was a comparison of OS on the intent-to-treat (ITT) population using a Bayesian posterior probability for the superiority of the combination over gemcitabine. Ninety-nine patients were planned, resulting in a frequentist design with ~60% power (1-sided, 0.2 type I error, no interim analysis) to detect a 2-month improvement in survival (7 months gemcitabine vs. 9 months LY2603618/gemcitabine). The Bayesian model [14, 15] incorporated historical gemcitabine data [16, 17] with prospective gemcitabine data to compare survival between the treatment arms and increase the power compared to the frequentist design. LY2603618 and gemcitabine would be considered superior to gemcitabine if the posterior probability of superiority exceeded 0.8. Simulation resulted in estimated power of 0.76 and type I error rate of 0.15. In addition to the Bayesian model, frequentist analysis of OS was also performed as a sensitivity analysis. The definition of secondary efficacy variables was consistent with standard conventions per RECIST (v 1.1) [18].

Exploratory analyses included: change from baseline in tumor size and carbohydrate antigen 19–9 (CA19-9) levels, and changes in QTcF from electrocardiograms (ECG) obtained at baseline and after LY2603618 administration on days 2 and 16 during cycle one.

### Safety

All patients who received at least one dose of study drug were evaluated for safety and toxicity. AE severity was graded using the Common Terminology Criteria for AEs (CTCAE) v 3.0.

### Pharmacokinetic/pharmacodynamic analysis

LY2603618 concentrations were quantified using a validated high-pressure liquid chromatography/mass spectrometry/mass spectrometry method. Whole blood samples were collected following the LY2603618 infusion on days 2 and 16 of cycle 1 before the start (<10 min) of infusion; immediately prior to the end of infusion (<5 min); and at 1, 3, and 24 h after the end of infusion. LY2603618 PK parameters were computed from the plasma concentration versus time data by standard noncompartmental analyses (Phoenix WinNonlin version 6.3, Pharsight, A Certara Company®; Princeton, NJ, USA). The PK parameters of maximum plasma concentration (C_max_) and area under the plasma concentration time-curve from time 0 to the time of the last measurable plasma concentration (AUC_[0-tlast]_) or infinity (AUC_[0-∞]_) on days 2 and 16 of cycle one were calculated, as well as the terminal elimination half-life (t_1/2_), volume of distribution at steady-state (V_ss_), systemic clearance (CL), percentage of AUC_[0-∞]_ extrapolated (%AUC_[tlast-∞]_), and the intra- and intercycle accumulation ratios (R_A_).

### Biomarker response

A nucleoside analog deoxyribonucleic acid (DNA) incorporation assay method measured the amount of gemcitabine incorporated into genomic DNA [19]. A sample for CA–19–9 analysis was collected at the start of each cycle.

## Results

### Patient disposition

Of the 107 enrolled patients, a total of 99 patients (*n* = 65, LY2603618/gemcitabine; *n* = 34, gemcitabine alone) were randomized and included in the ITT population. The first patient was enrolled on 26 February 2009. Patient demographics and disease characteristics at baseline are summarized in Table 1. The majority of the patient population (mean age, 64 years) presented with Stage IV disease (76.8%) and more than 90% had an ECOG PS 0–1 at study entry (Table 1). The primary reasons for study discontinuation in the LY2603618/gemcitabine arm and gemcitabine alone arm, respectively, included: progressive disease (70.8%; 61.8%), AE (12.3%; 17.6%), subject decision (4.6%; 14.7%), investigator decision (6.2%; 2.9%), death (4.6%; 2.9%), and protocol violation (1.5%; 0).

**Table 1:** Patient demographics and disease characteristics at baseline

Parameter	LY2603618/gemcitabine	Gemcitabine
	(*n* = 65)	(*n* = 34)
Age, years		
Mean (SD)	64.3 (8.3)	64.4 (10.1)
Median	64.0	65.5
Range	47–83	39–90
Gender, n (%)		
Female	23 (35.4)	14 (41.2)
Male	42 (64.6)	20 (58.8)
Race, n (%)		
White	62 (95.4)	32 (94.1)
Black or African American	2 (3.1)	2 (5.9)
American Indian or Alaska Native	1 (1.5)	0
BSA at baseline (m^2^)		
Mean (SD)	1.8 (0.2)	1.8 (0.2)
Median	1.8	1.7
Range	1.3–2.5	1.4–2.5
Disease stage, n (%)		
II	6 (9.2)	3 (8.8)
III	8 (12.3)	5 (14.7)
IV	50 (76.9)	26 (76.5)
Unknown	1 (1.5)	0
ECOG PS, n (%)		
0	28 (43.1)	14 (41.2)
1	31 (47.7)	17 (50)
2	6 (9.2)	3 (8.8)

*BSA* body mass index; *ECOG PS* Eastern Cooperative Oncology Group performance status; *LY2603618/gemcitabine* LY2603618 (230 mg flat dose) combined with gemcitabine 1000 mg/m^2^; *m* ^*2*^ meters squared; *mg* milligrams; *n* number of patients; *SD* standard deviation

### Clinical efficacy

**Fig. 1:**
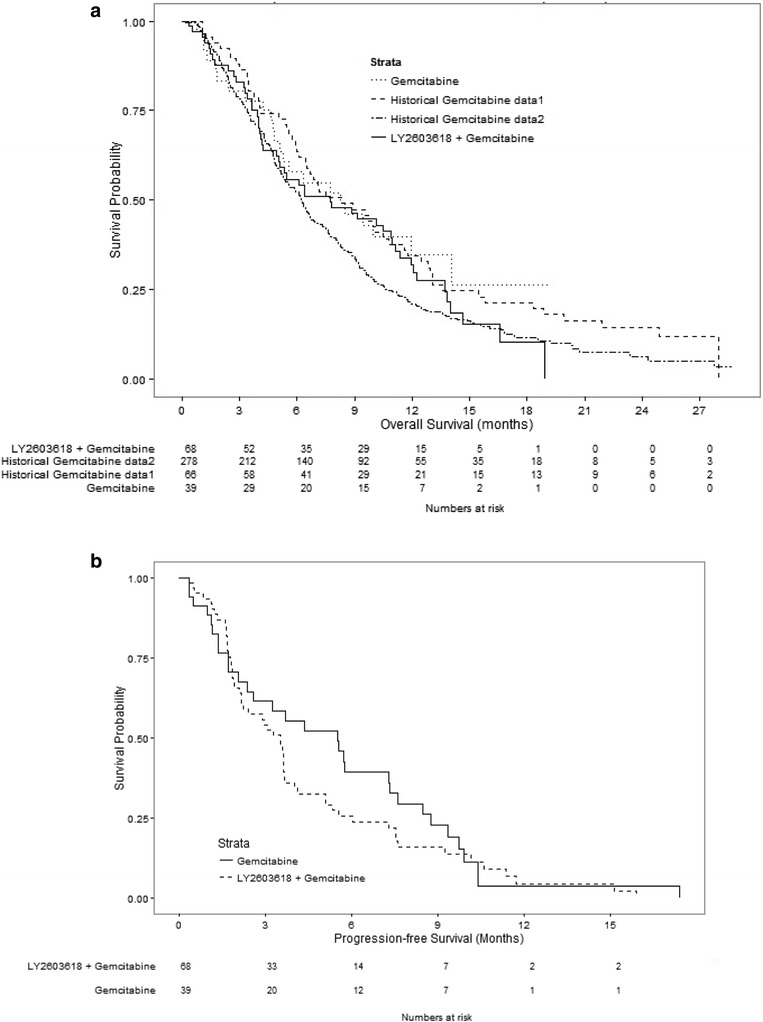
**a** Overall survival. Kaplan Meier survival curves of patients treated with LY2603618/gemcitabine combination therapy compared with historical gemcitabine studies. [^1^Jänne PA, Paz-Ares L, Oh Y, Eschbach C, Hirsh V, Enas N, Brail L, von Pawel J. Randomized, double-blind, phase II trial comparing gemcitabine-cisplatin plus the LTB4 antagonist LY293111 versus gemcitabine-cisplatin plus placebo in first-line non-small-cell lung cancer. J Thorac Oncol. 2014;9:126–31. ^2^Oettle H, Richards D, Ramanathan RK, van Laethem JL, Peeters M, Fuchs M, Zimmerman A, John W, Von Hoff D, Arning M, Kindler HL. A phase III trial of pemetrexed plus gemcitabine versus gemcitabine in patients with unresectable or metastatic pancreatic cancer. Ann Oncol. 2005;16:1639–45.] **b** Progression-free survival. Kaplan Meier survival curves of patients treated with LY2603618/gemcitabine compared with gemcitabine monotherapy

The Bayesian model was applied to compare OS between treatments. The posterior probability of superiority of LY2603618/gemcitabine over gemcitabine alone was 0.33, which did not exceed the pre-specified threshold of 0.8. These findings were confirmed by the frequentist analysis. The median OS was 7.8 months (range, 0.3–18.9 months) for LY2603618/gemcitabine and 8.3 months (range, 0.8–19.1+ months) for gemcitabine alone (Fig. 1a).

Overall, LY2603618/gemcitabine was not statistically superior to gemcitabine alone when PFS, duration of response, ORR, and clinical benefit rate were assessed (Table 2). The median PFS was 3.5 months (range, 0–15.9 months) for LY2603618/gemcitabine and 5.6 months (range, 0–17.4 months) for gemcitabine (Table 2; Fig. 1b). No complete response (CR) was observed with either treatment. Although not statistically significant, a numerically higher percentage of patients had a partial response (PR) in the LY2603618/gemcitabine arm (21.5%; 95% confidence intervals [CI], 12.3–33.5) than in the gemcitabine arm (8.8%; 95% CI, 1.9–23.7). No distinguishing baseline characteristics were noted among patients with a response. Due to the overlapping confidence intervals, the clinical significance of the difference in response rates is unknown. The clinical benefit rate was 55.4% (95% CI, 42.5–67.7) and 64.7% (95% CI, 46.5–80.3) in the LY2603618/gemcitabine and gemcitabine arms, respectively. No significant difference in the change in tumor size (the sum of target lesions, per RECIST) from baseline to 8 weeks was observed between treatments (*P* = .6726).

**Table 2:** Secondary efficacy endpoints

	LY2603618/gemcitabine	Gemcitabine
	(*n* = 65)	(*n* = 34)
Progression-free survival, mos.		
Median (range)	3.5 (0–15.9)	5.6 (0–17.4)
Duration of response, mos.		
Median (range)	3.5 (1.5–14.1)	6.0 (3.7–6.8)
Best Overall Response, n (%; 95% CI)		
CR	0	0
PR	14 (21.5%; 12.3–33.5)	3 (8.8%; 1.9–23.7)
SD	22 (33.8%; 22.6–46.6)	19 (55.9%; 37.9–72.8)
Clinical Benefit Rate, n (%; 95% CI)	36 (55.4%; 42.5–67.7)	22 (64.7%; 46.5–80.3)

*CI* confidence interval; *CR* complete response; *LY2603618/gemcitabine* LY2603618 (230 mg flat dose) combined with gemcitabine 1000 mg/m^2^; *mos* months; *n* number of patients; *PR* partial response; *SD* stable disease

### Safety

The median number of cycles completed was 2.0 (range, 0–16 cycles) for LY2603618/gemcitabine and 2.5 (range, 0–18 cycles) for gemcitabine. As shown in Table 3, study drug-related treatment-emergent AEs (TEAEs) were comparable between LY2603618/gemcitabine (89.2%) and gemcitabine (91.2%). The most frequently observed TEAEs in both arms were nausea, thrombocytopenia, fatigue, and neutropenia. Fewer patients experienced anemia with LY2603618/gemcitabine (13.8%) than with gemcitabine (26.5%). In contrast, a higher incidence of vomiting, decreased appetite, and stomatitis was observed for LY2603618/gemcitabine than for gemcitabine. For each arm, neutropenia and thrombocytopenia were the most common grade 3/4 TEAEs possibly related to treatment, in addition to anemia, which was also common to gemcitabine (Table 4). Serious adverse events (SAE) related to study treatment were reported among 13.8% and 23.5% of patients in the LY2603618/gemcitabine and gemcitabine arms, respectively.

**Table 3:** Study drug-related treatment-emergent adverse events in ≥10% of the safety population

Preferred Term, n (%)	LY2603618/gemcitabine	Gemcitabine
	(*n* = 65)	(*n* = 34)
Patients with ≥1 TEAE	58 (89.2)	31 (91.2)
Thrombocytopenia	21 (32.3)	14 (41.2)
Nausea	22 (33.8)	8 (23.5)
Fatigue	16 (24.6)	10 (29.4)
Neutropenia	14 (21.5)	9 (26.5)
Anemia	9 (13.8)	9 (26.5)
Vomiting	14 (21.5)	3 (8.8)
Decreased appetite	12 (18.5)	3 (8.8)
Diarrhea	11 (16.9)	3 (8.8)
Pyrexia	9 (13.8)	5 (14.7)
Asthenia	7 (10.8)	5 (14.7)
Constipation	9 (13.8)	3 (8.8)
Leukopenia	7 (10.8)	5 (14.7)
Stomatitis	10 (15.4)	1 (2.9)
Alopecia	6 (9.2)	4 (11.8)
Oedema peripheral	7 (10.8)	2 (5.9)

*LY2603618/gemcitabine* LY2603618 (230 mg flat dose) combined with gemcitabine 1000 mg/m^2^; *n* number of patients; *TEAE* treatment-emergent adverse events

**Table 4:** Grade 3/4 study drug-related treatment-emergent adverse events in ≥5% of the safety population

Preferred Term, n (%)	LY2603618/gemcitabine (*n* = 65)		Gemcitabine (*n* = 34)	
	Grade 3	Grade 4	Grade 3	Grade 4
Patients with ≥1 TEAE	25 (38.5)	6 (9.2)	19 (55.9)	3 (8.8)
Decreased hemoglobin	2 (3.1)	0	4 (11.8)	0
Decreased leukocytes	5 (7.7)	0	1 (2.9)	1 (2.9)
Decreased neutrophils/				
Decreased platelets	7 (10.8)	0	3 (8.8)	1 (2.9)
Thrombotic microangiopathy	0	0	2 (5.9)	0
Fatigue	1 (1.5)	1 (1.5)	3 (8.8)	0
Dehydration	0	0	2 (5.9)	0
Hyponatremia	2 (3.1)	0	2 (5.9)	0

*LY2603618/gemcitabine* LY2603618 (230 mg flat dose) combined with gemcitabine 1000 mg/m^2^; *n* number of patients; *TEAE* treatment-emergent adverse events

Fourteen patients (*n* = 8, LY2603618/gemcitabine; *n* = 6, gemcitabine) discontinued the study due to AEs. Of the eight patients who discontinued in the LY2603618/gemcitabine arm, four events (grade 4 cerebrovascular accident, grade 1 left bundle branch block, grade 3 acute pulmonary oedema, and grade 3 atrial fibrillation) were possibly related to treatment. Of the six patients who discontinued in the gemcitabine arm, four possibly related events occurred (grade 3 thrombotic microangiopathy, grade 4 acute renal failure, grade 2 thrombocytopenia, and grade 3 hemolytic uraemic syndrome). Four deaths were reported during the study; three due to disease progression and one due to a non-related peripheral arterial ischemia event.

### Pharmacokinetic/pharmacodynamic analyses

LY2603618 demonstrated consistent PK parameters after single (day 2) and repeat administration (day 16) during cycle 1 (Table 5). The LY2603618 plasma systemic exposure targets (i.e., AUC_(0-∞)_ ≥21,000 ng hr/mL and C_max_ ≥2000 ng/mL) that correlate with maximal pharmacodynamic (PD) effect observed in nonclinical HT-29 xenograft models (data on file) were achieved on a mean cohort basis after 230 mg of LY2603618 (Table 5). More specifically, 87% and 73% of the individual PK profiles on days 2 and 16 of cycle 1 were above the targets for C_max_ and AUC_(0-∞)_, respectively.

**Table 5:** Summary of LY2603618 noncompartmental pharmacokinetic parameter estimates

Parameter	Geometric Mean (CV%) 230 mg LY2603618	
	Cycle 1	
	Day 2	Day 16
	(*n* = 58)	(*n* = 48)
C_max_ (ng/mL)	3170 (50)	3410 (50)
t_max_^a^ (h)	1.00 (0.88–1.38)	1.00 (0.88–1.83)
C_av,24_ (ng/mL)	966 (68) ^d^	987 (60) ^e^
AUC_(0–24)_ (ng*h/mL)	23200 (68) ^d^	23700 (60) ^e^
AUC_(0-∞)_ (ng*h/mL)	29400 (84) ^d^	29100 (74) ^e^
AUC_(tlast-∞)_ (%)	14.3 (131) ^d^	12.0 (152) ^e^
CL (L/h)	7.79 (84) ^d^	7.87 (74) ^e^
V_ss_ (L)	104 (48) ^d^	95.1 (42) ^e^
t_1/2_ (h)	9.67 (48) ^d^	8.86 (48) ^e^
R_A_^b^	–	108 (32) ^f^
R_A_^c^	–	–

*AUC*_*(0 - ∞)*_ area under the plasma concentration time-curve from time 0 to infinity; *AUC*_*(0–24)*_ area under the plasma concentration time-curve from time 0 to 24 h; *AUC*_*(tlast - ∞)*_ fraction of AUC_(0 - ∞)_ extrapolated from the time of the last measurable plasma concentration (t_last_) to infinity; *C*_*av,24*_ average plasma concentration over 24 h calculated using AUC_(0–24)_; *CL* systemic clearance; *C*_*max*_ maximum plasma concentration; *CV%* percent coefficient of variation; *m* ^*2*^ meters squared; *mg* milligrams; *n* number of pharmacokinetic observations; *NC* not calculated; *R*_*A*_ accumulation ratio; *t*_*max*_ time of maximum observed plasma concentration; *V*_*ss*_ volume of distribution at steady state following intravenous (IV) administration; *t*_*1/2*_ elimination half-life

^a^Median (range)

^b^Intracycle accumulation ratio [Cycle 1 Day 16 AUC_(0 - ∞)_/Cycle 1 Day 2 AUC_(0 - ∞)_]

^c^Intercycle accumulation ratio [Cycle 2 Day 2 AUC_(0 - ∞)_/Cycle 1 Day 2 AUC_(0 - ∞)_]

^d^ *n* = 54

^e^ *n* = 42

^f^ *n* = 38

For the PD analyses, dFdC was incorporated into DNA following gemcitabine administration, with the levels declining to almost baseline by the end of each treatment cycle. The highest levels of dFdC incorporation were observed on days 8 and 15 across all doses. The increases in the amount of dFdC incorporation did not correspond to increasing doses of LY2603618.

Of the patients who had baseline CA19-9 levels > upper limit of normal, a similar percentage of patients (65.4% LY2603618/gemcitabine; 64% gemcitabine) experienced a >50% reduction from baseline in CA19-9 levels.

### QTcF assessment

In a time-point exploratory QTcF assessment, no clinically significant trends in ECG parameter changes were reported. Five patients had a change in QTcF from baseline between 30 and 60 milliseconds (msec); no patients had a change in QTcF >60 msec.

## Discussion

The current study was part of a Phase I/II study designed to compare the OS of LY2603618/gemcitabine to gemcitabine alone. This study used a Bayesian augmented control design to incorporate historical gemcitabine data, which minimized the number of patients needed for the treatment to be evaluated. Stage II or III patients not amenable to resection with curative intent or Stage IV disease were included in the current study to match the populations used in the historical studies used as reference data. The OS of LY2603618/gemcitabine was not superior to gemcitabine alone in patients with locally advanced or metastatic pancreatic cancer by either the Bayesian or frequentist approach. In addition, no significant differences between arms in any of the secondary endpoints were observed.

The safety profiles were comparable between arms, indicating that the addition of LY2603618 did not significantly change the safety profile of gemcitabine. This is consistent with the CHK1 inhibitor MK8776 [20], but in contrast to the data reported with the CHK1 selective inhibitors GDC-0425 and AZD7762 [21, 22]. In a Phase 1 study with AZD7762, unpredictable cardiac toxicity was observed [21]. Although it was demonstrated safe and feasible to administer GDC-0425 with gemcitabine, the CHK1 inhibitor appeared to increase some of the toxicities associated with gemcitabine [22].

A trend towards a lower LY2603618 systemic exposure and more rapid CL associated with a larger interpatient variability in Phase II (Table 5) compared to Phase I was observed [13]. This is likely a result of the more limited PK sampling schedule (sampling to only 24 h post dose) used in the Phase II study (i.e., larger AUC_(tlast-∞)_ (%) values; Table 5), thereby limiting the capability of the conventional PK analysis method to accurately quantify the terminal elimination phase of LY2603618 and resulting in an underestimate of AUC_(0-∞)_ and overestimate of CL. In contrast, the LY2603618 PK profiles over the first 24 h from Phase II demonstrated a high degree of concordance with the PK profiles from Phase I. The average t_1/2_ following administration of 230 mg LY2603618 was consistent with a t_1/2_ suitable for achieving and maintaining the desired target human exposures while minimizing the intra- and intercycle accumulation (Table 5). Gemcitabine did not appear to affect the PK of LY2603618, as the PK parameters reported in this study were similar to the PK parameters calculated after LY2603618 monotherapy [23].

The study had inherent limitations that may have contributed to the negative clinical outcome observed. In addition, due to the lack of a clinically-validated PD marker to quantify direct CHK1 inhibition by LY2603618, the magnitude and duration of CHK1 target inhibition at 230 mg is neither known nor has it been correlated to clinical responses. Therefore, it is possible the PK surrogate targets (i.e., AUC_(0-∞)_ ≥21,000 ng•hr/mL and C_max_ ≥2000 ng/mL) derived from nonclinical xenograft models for maximal PD response were not appropriate thresholds to predict clinical responses in humans. In addition, inclusion of only patients with Stage IV disease may have yielded a more favorable clinical outcome.

One Phase III randomized trial comparing gemcitabine with FOLFIRINOX reported statistically significant improvements in OS (hazard ratio [HR] 0.57, *P* < .0001), PFS (HR 0.47, *P* < .0001), and ORR (*P* = .0001) in chemo-naïve patients with ECOG PS 0 and 1 [5]. Despite the clincial efficacy observed with FOLFIRINOX, this treatment was associated with more frequent and more severe toxicity [5]. As a result, only patients with an adequate PS are typically eligible for FOLFIRINOX treatment. Recent meta-analyses have reported that patients with poorer performance had less OS benefit from combined therapies for metastatic pancreatic cancer [24, 25]. Since FOLFIRINOX emerged as a treatment option during the conduct of this study, there was concern that a greater proportion of patients with low PS status who were not eligible for FOLFIRINOX would be included. However, only 9.2% and 8.8% of patients on the experimental arm and control arm, respectively, were PS 2 and so this consideration was unlikely to have affected the outcome.

The Metastatic Pancreatic Adenocarcinoma Clinical Trial (MPACT) demonstrated improved clinical efficacy with gemcitabine/*nab*-paclitaxel than gemcitabine (median OS, 8.5 months vs. 6.7 months, respectively); however, fatigue, neuropathy, and neutropenia were more common among patients receiving combination therapy than monotherapy [4, 8]. Interestingly, the overall incidence of grade III/IV study drug-related TEAEs in the current study was not increased in the LY2603618/gemcitabine arm compared with the gemcitabine arm, except for the incidence of grade 3 leukocytes and platelets, which was higher in the LY2603618/gemcitabine arm.

## Conclusion

OS was not improved with the addition of LY2603618/gemcitabine compared with gemcitabine alone. The safety and PK profiles were comparable between treatment arms. As a result of this finding, LY2603618/gemcitabine will not be further developed for the treatment of patients with pancreatic cancer.

## Abbreviations

%AUC_[tlast-∞]_: Percentage of AUC_[0-∞]_ extrapolated
5-FU: Fluorouracil
AE: Adverse event
AUC_[0-tlast]_: Area under the plasma concentration time-curve from time 0 to the time of the last measurable plasma concentration
AUC_[0-∞]_: Area under the plasma concentration time-curve from time 0 to infinity
AUC_(0–24)_: Area under the plasma concentration time-curve from time 0 to 24 h
AUC_(tlast - ∞)_: Fraction of AUC_(0 - ∞)_ extrapolated from the time of the last measurable plasma concentration (t_last_) to infinity
CA19-9: Carbohydrate antigen 19–9
C_av,24_: Average plasma concentration over 24 h calculated using AUC_(0–24)_
CHK1: Checkpoint kinase 1
CI: Confidence intervals
CL: Systemic clearance
C_max_: Maximum plasma concentration
CR: Complete response
CTCAE: Common terminology criteria for adverse events
CV%: Percent coefficient of variation
dFdC 2’: 2’-difluorodeoxycytidine
DNA: Deoxyribonucleic acid
ECG: Electrocardiogram
ECOG PS: Eastern cooperative oncology group performance status
FOLFIRINOX: Oxaliplatin, irinotecan, leucovorin, and 5-FU
ITT: Intent-to-treat
m^2^: Meters squared
mg: Milligram
MPACT: Metastatic pancreatic adenocarcinoma clinical trial
msec: Millisecond
NC: Not calculated
ORR: Overall response rate
OS: Overall survival
PD: Pharmacodynamic
PFS: Progression-free survival
PK: Pharmacokinetics
PR: Partial response
PS: Performance status
QTcF: Fridericia’s heart rate-corrected QT interval
R_A_: Intra- and intercycle accumulation ratios
RNAi: Ribonucleic acid interference
SAE: Serious adverse event
t_1/2_: Terminal elimination half-life
TEAE: Treatment-emergent AEs
t_max_: Time of maximum observed plasma concentration
V_ss_: Volume of distribution at steady-state
